# Supplementary material for: Tesla valves and capillary structures-activated thermal regulator
Source: Nat Commun. 2023 Jul 6;14:3996. doi: 10.1038/s41467-023-39289-5 (PMC10325955; doi:10.1038/s41467-023-39289-5)
Supplement: Supplementary file 1 — Supplementary Information [file 41467_2023_39289_MOESM1_ESM.pdf]

Tesla valves and capillary structures-activated thermal regulator

Wenming Li[1], Siyan Yang[2,3], Yongping Chen[1,4,*], Chen Li[5] and Zuankai Wang[2,*]

[1]Key Laboratory of Energy Thermal Conversion and Control of Ministry of Education, School of Energy and Environment, Southeast University, Nanjing, 210096, PR China

[2]Department of Mechanical Engineering, Hong Kong Polytechnic University, Hong Kong, PR China

[3]Department of Mechanical Engineering, City University of Hong Kong, Hong Kong, PR China

[4]Jiangsu Key Laboratory of Micro and Nano Heat Fluid Flow Technology and Energy Application, School of Environmental Science and Engineering, Suzhou University of Science and Technology, Suzhou, 215009, PR China

[5]Department of Mechanical Engineering, University of South Carolina, Columbia, SC, USA

*Corresponding e-mail: ypchen@seu.edu.cn (Y.C.) and zk.wang@polyu.edu.hk (Z.W.)

1. Supplementary figures

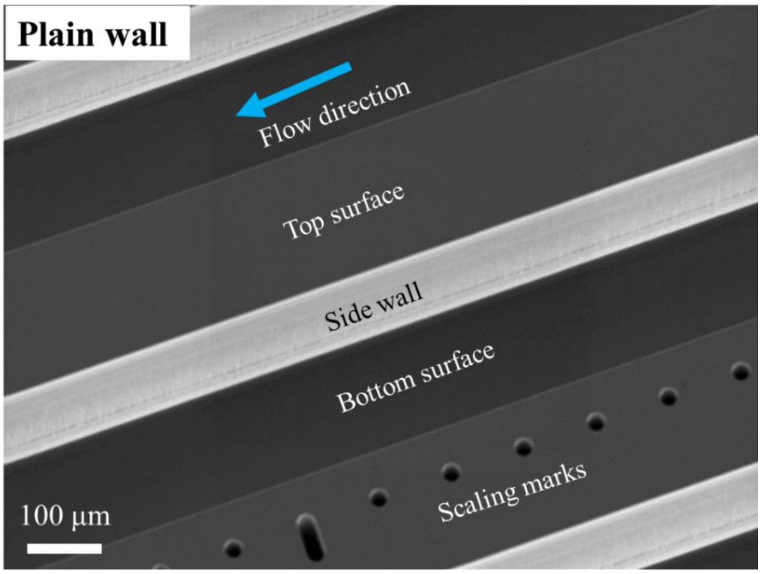

Supplementary Fig. 1 Configuration of conventional plain wall microchannels. Five parallel plain wall microchennels were selected as one of control devices for comparison. The dimensions of this configuration are (length, width and height) 10 mm, 200 μm and 250 μm, respectively.

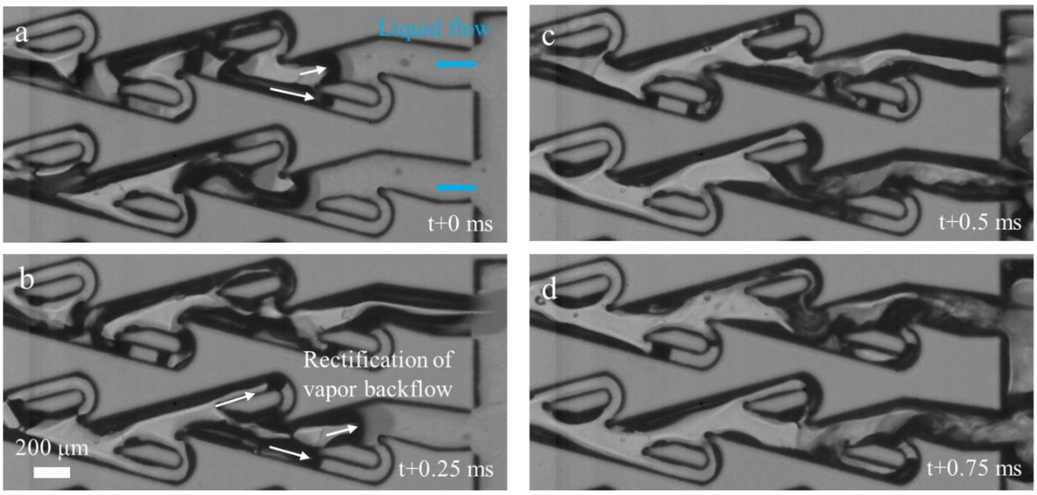

Supplementary Fig. 2 Successful rectification of two-phase backward flow at switched-on state. An extended view of the dynamic two-phase behaviors near the inlet. The two-phase backward flow is gradually rectified by the periodically distributed Tesla valves under a heat flux of 354 W cm^{-2} at $\dot{m}$ = 0.18 kg h^{-1}.

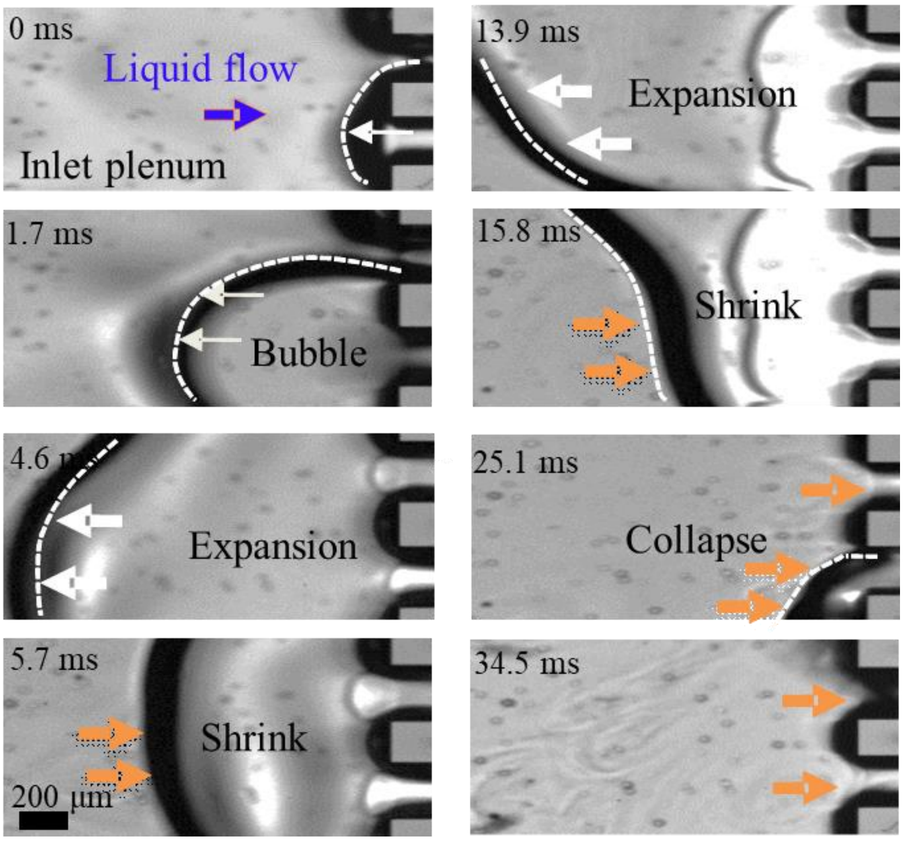

Supplementary Fig. 3 Bubbly dynamics near the inlet plenum of conventional plain wall microchannels. Time-elapsed optical images recorded by a high-speed camera showing the bubble dynamics including bubble expansion, shrinkage, and collapse processes in the inlet under a heat flux of 150 W cm^{-2} at $\dot{m}$ = 0.303 kg h^{-1}. Upon nucleation on the sidewall, bubbles grow in individual channels and experience a transient oscillation process including expansion (0 - 4.6 ms), shrinkage (4.6 - 5.7 ms), expansion (5.7 - 13.9 ms), and eventual collapse (15.8 - 25.1 ms). Apparent vapor

backflows are also observed between 4.6 ms and 13.9 ms due to the non-equilibrium nature of the two-phase heat transfer process. Moreover, a persistent vapor column blocks the liquid refreshing downstream channels rapidly. As a result, a large dry-out area in the downstream channels is formed, leading to the unwanted liquid flow crisis.

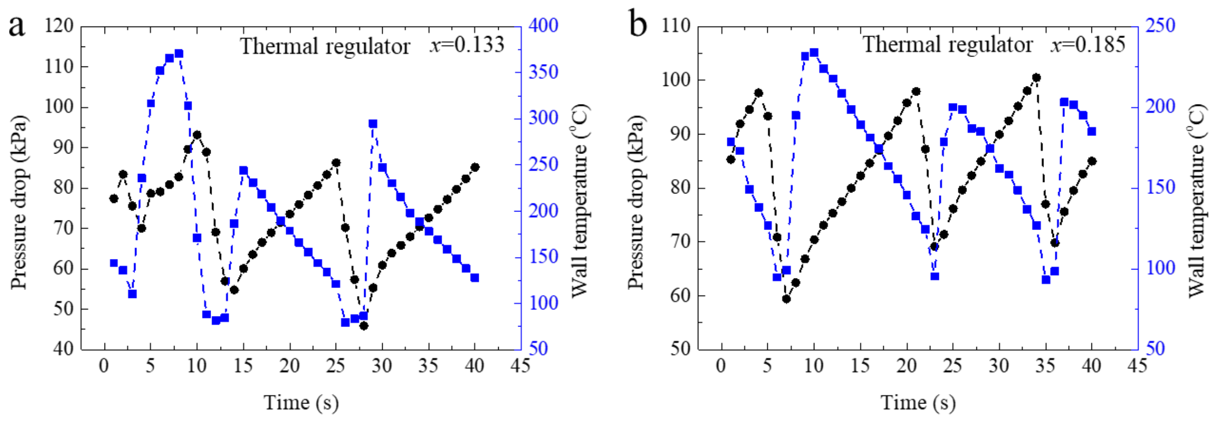

Supplementary Fig. 4 Characteristics of pressure drop and wall temperature periodic oscillations. Pressure drop and temperature oscillations are plotted in a short duration of 40 s at two different exit vapor qualities of 0.133 and 0.185, respectively.

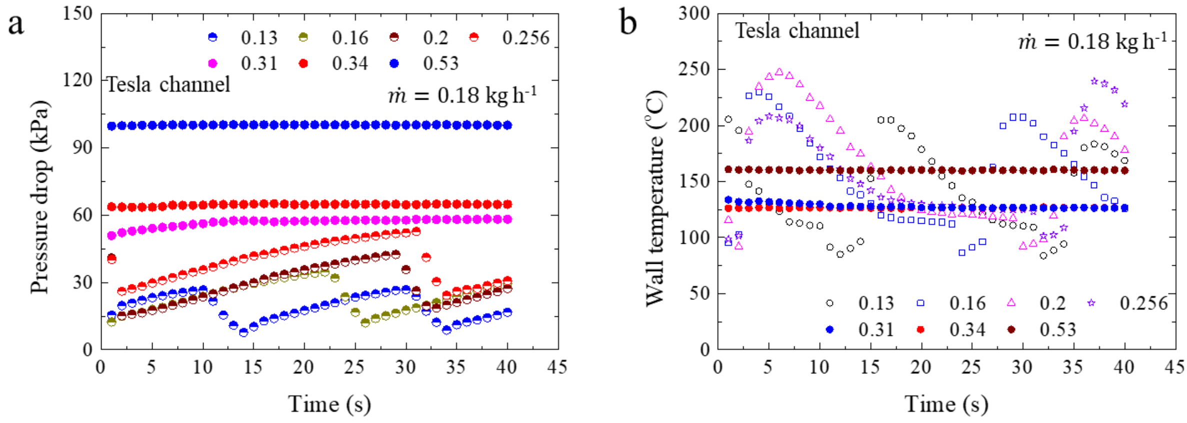

Supplementary Fig. 5 Two distinct working states of Tesla channel in the forward direction. The variation of pressure drop and wall temperature at χ ranging from 0.13 to 0.53 under $\dot{m}$ = 0.18 kg h^{-1}.When χ < 0.3, both the pressure drop and the wall temperature fluctuate periodically whereas they become almost constant when χ > 0.25, suggesting the switched-on Tesla channels.

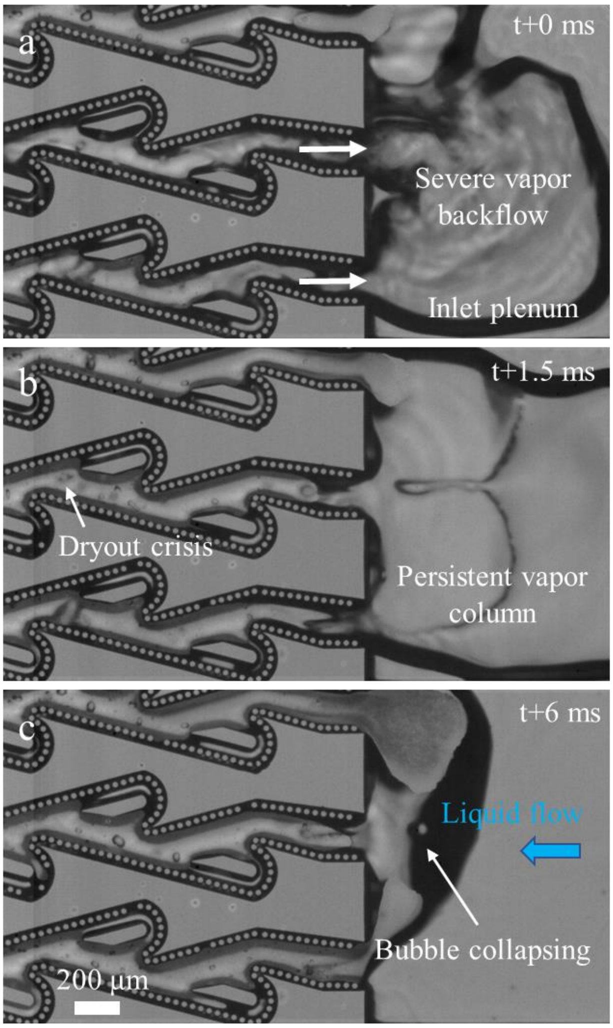

Supplementary Fig. 6 Bubble dynamics near the inlet plenum of our thermal regulator. When the thermal regulator is in the switched-off state, a persistent vapor column is observed near the inlet under a heat flux of 248 W cm^{-2} at $\dot{m} = 0.303$ kg h^{-1}.

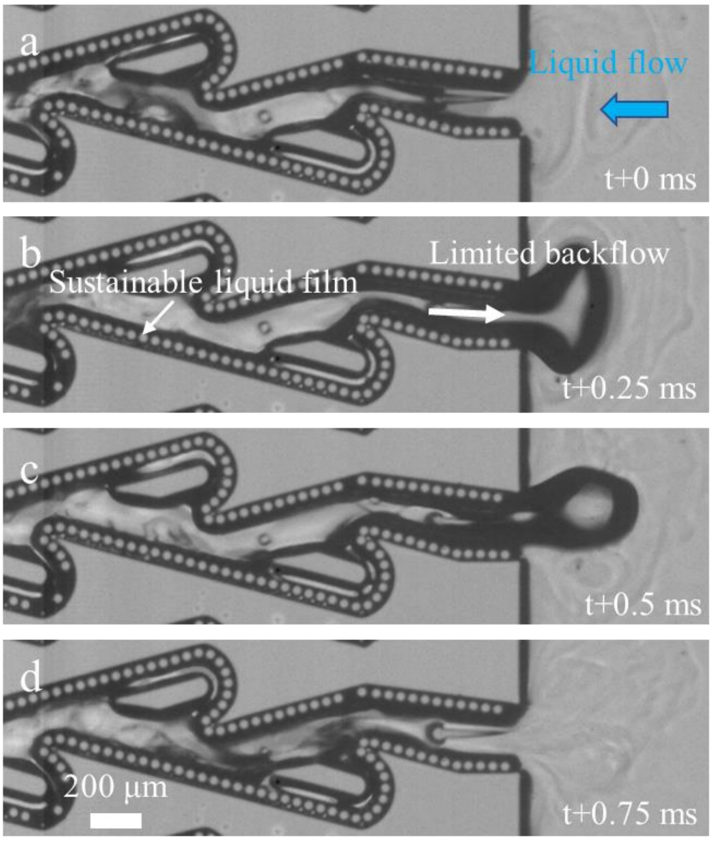

Supplementary Fig. 7 Achievement of directional two-phase flow. When the diode is switched on at $\chi > 0.25$, vapor columns collapses quickly and then liquid timely reflushes to prevent the potential local dry-out inside the channels under a heat flux of 363 W cm^{-2} at $\dot{m} = 0.303$ kg h^{-1}.

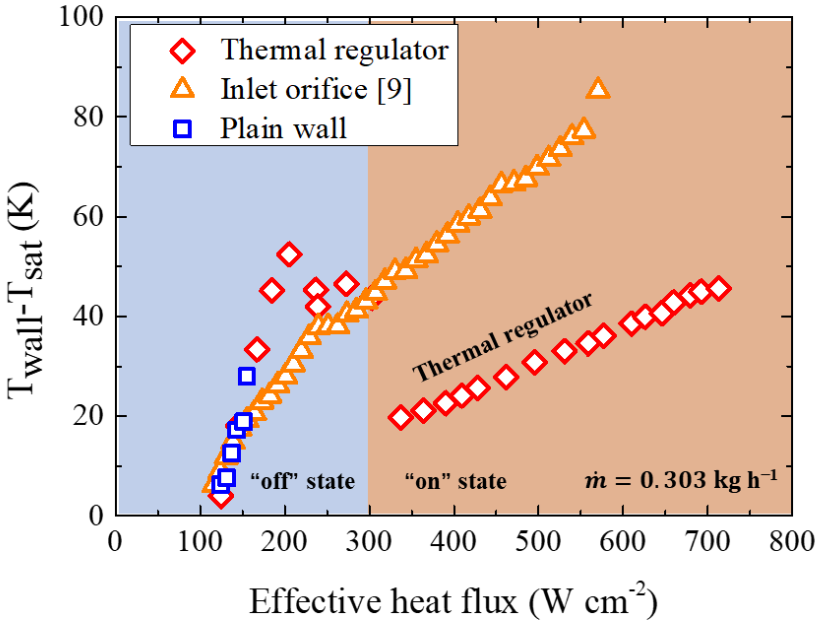

Supplementary Fig. 8 Controlling wall temperature enabled by thermal regulator at high heat fluxes.

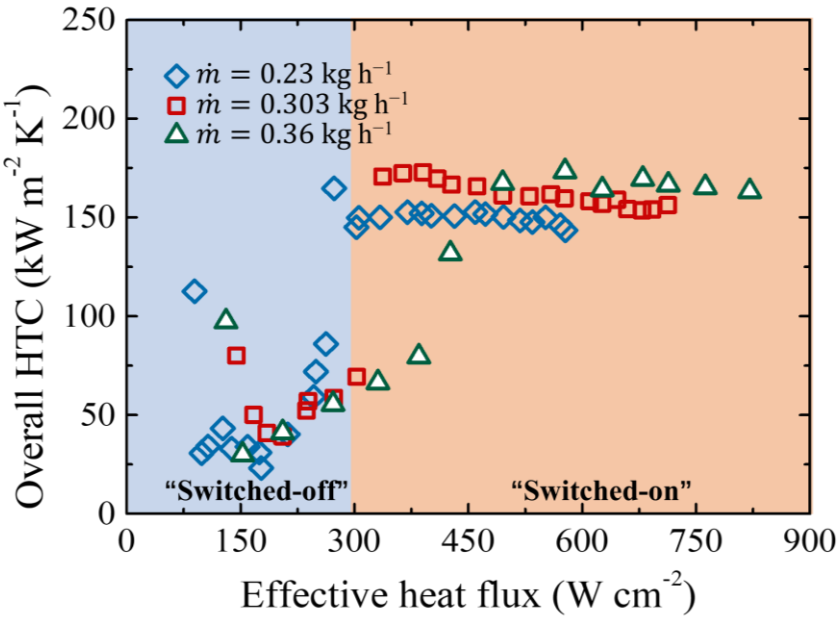

Supplementary Fig. 9 High overall HTC in the switched-on state in forward direction. The overall HTC curves under flow rates ranging from 0.23 kg h^{-1} to 0.36 kg h^{-1}, which displays a unique "$\sqrt{\ }$" shape. In the switched-off state, the HTC sharply declines to ~30 kW m^{-2} K^{-1} after the onset of boiling whereas a high HTC as high as ~175 kW m^{-2} K^{-1} is achieved in the switched-on state.

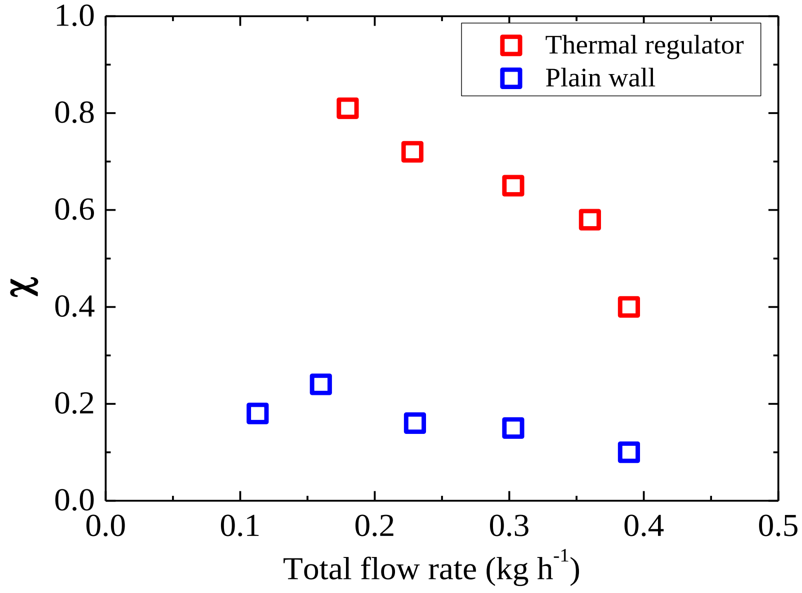

Supplementary Fig. 10 High χ in our thermal regulator. With the synergistic effect of Tesla valves and capillary fences, the χ is up to 0.8 at $\dot{m}$ = 0.18 kg h^{-1}, nearly 4-fold larger than that of conventional device, suggesting the potential maxima of boiling heat transfer.

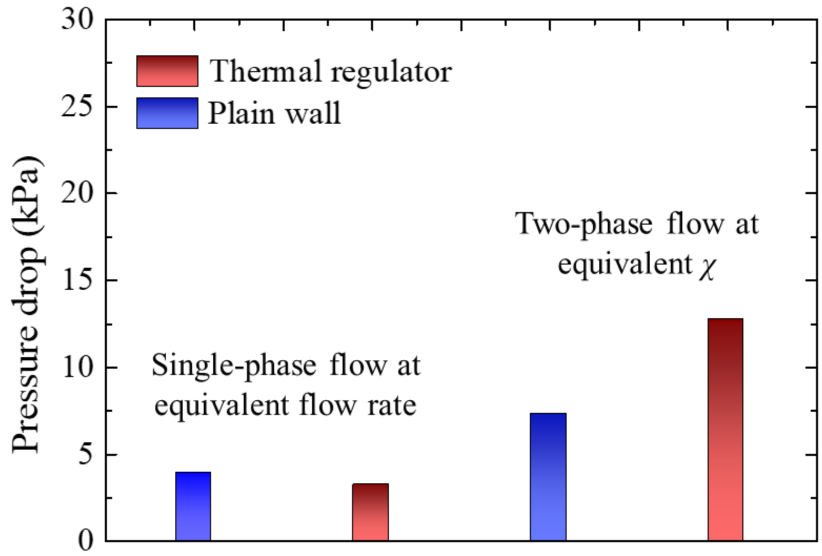

Supplementary Fig. 11 High thermal performance without the sacrifice of pressure drop. The pressure difference Δ*p* between the thermal regulator and conventional device is insignificant in both single-phase and two-phase flow regions.

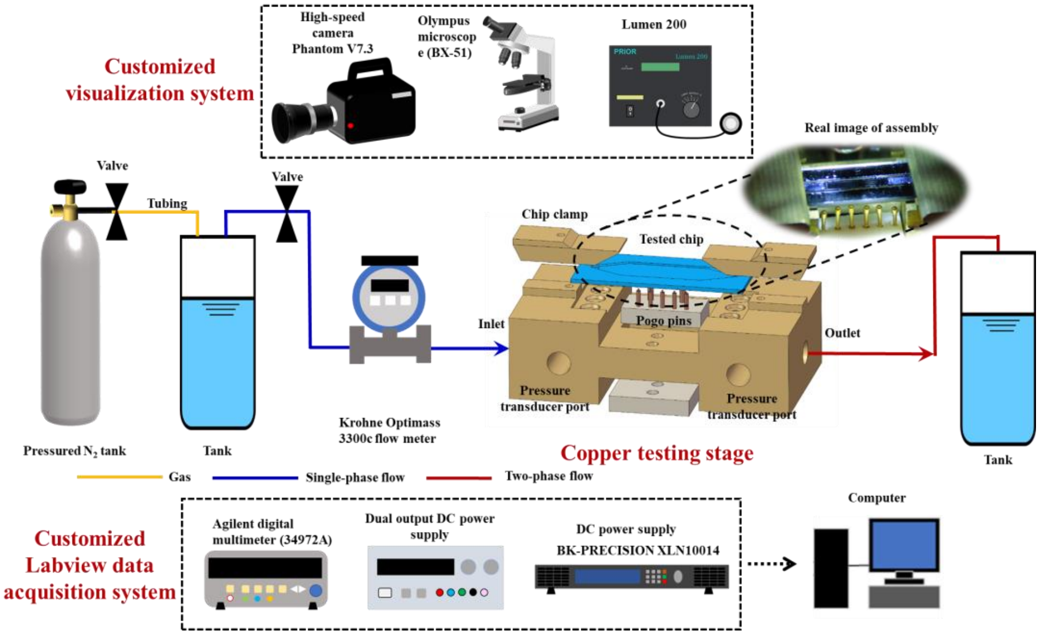

Supplementary Fig. 12 Experimental setup. The major components of the experimental setup, which includes an optical imaging system, a data acquisition unit, and an open coolant loop.

2. Supplementary tables

Table S1 The nonlinear and switchable heat transfer performance of selected thermal diodes, thermal switches and thermal regulators in convection.

Applications	Working principle	Thermal switches and regulators	Thermal diodes	References
		On/off ratio $r = G_{on}/G_{off}$	Rectification $\gamma = \frac{G_{fwd} - G_{rev}}{G_{rev}}$	
Jumping droplet thermal diodes	Controlling droplets jumping by changing properties of surfaces	2	150	[1] and [2]
Thermal switch	Manipulating liquid droplets by electro-wetting	2.5-15	N/A	[3]
	Controlling the contact angle of liquid droplet	2.4 and 4	N/A	[4]
Thermal valve	Actuating magnetic nanofluids by a non-uniform magnetic field	15	N/A	[5]
Vapor chamber	Returning self-propelled jumping drops to the evaporator	23	N/A	[6]
	Driving working fluid circulation in one-way by surface tension force	2	N/A	[7]
Film boiling	Suppressing Leidenfrost state using AC electric fields	5-20	N/A	[8,9]
Pooling boiling	Electric field to suppress film boiling	10	N/A	[10]
Flow boiling in Tesla channels	Regulating boiling heat transfer by shaping two-phase transport	4.2	1	Current study
Flow boiling in thermal regulator	Regulating boiling heat transfer by shaping two-phase transport and enhancing thin film evaporation	6	N/A	Current study

Table S2 Comprehensive comparison of normalized boiling heat transfer performances in microchannels on DI-water.

Geometry configuration	Hydraulic diameter, D_h (μm)	Flow rate (ml min^{-1}/ kg m^{-2}s^{-1})	CHF (W cm^{-2})	q''_{CHF}/ $q''_{CHF-base}$	h_{HTC}/$h_{HTC\text{-}base}$
Microchannels with micropinfin fences [11]	234	1.2-5.8/80-389	270-830	5.4	3
Wicked single channel [12]	500	4.5/300	969	1.6	1.7
Staggered micropin fin heat sink [13]	439	3.1-7.1/346-794	20-350	N/A	N/A
Microchannels installed with inet orifices [14]	223	1.2-4.5/83-303	142-440	3.5	1.5
Diamond microchannels with microporous copper [15]	N/A	60, 186/-	820, 1280	N/A	N/A
Gradient wick channels [16]	1536	-/140-340	870	1.6	1.6
Combination of micronozzles and cavities [17]	234	3.75-10.2/250-680	420-1016	3.9	2.2
Current study	223	3-6/186-389	515-835	6	5

Table S3 Summary of maximum HTC at CHF conditions flow boiling.

Geometry configuration	CHF (W cm^{-2})	Max. HTC
Microchannels with micropinfin fences [11]	830	150
Staggered micropin fin heat sink [13]	350	70
Microchannels installed with inet orifices [14]	440	40
Diamond microchannels with microporous copper [15]	1340	320
Gradient wick channels [16]	870	180
Combination of micronozzles and cavities [17]	1016	109
**Thermal regulator**	835	175
**Tesla channel**	650	100
Plain microchannel [18]	150	35
Roughened channels [19]	270	80

Tapered gap [20]	1060	280
Reentrant cavity [14]	643	55

Table S4 Decoupling the effects of Tesla bend and Tesla Island on thermal performance.

<table>
<tr><th>Tesla type geometries</th><th>Enhanced CHF and HTC in forward direction</th><th>Mechanisms of enhancement</th></tr>
<tr><td>Copper saw-like microchannel configuration
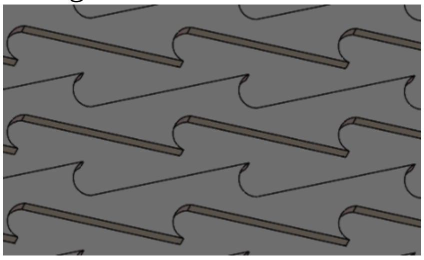</td><td>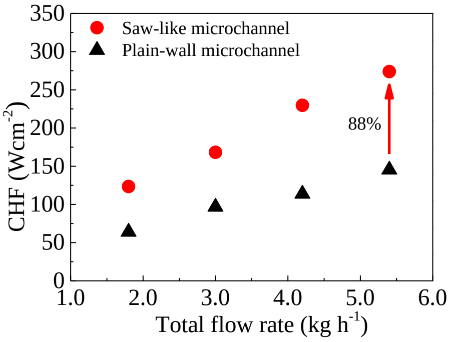

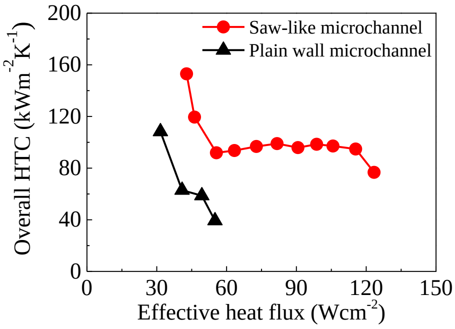
</td><td>CHF is significantly enhanced, about 88%, by suppressing vapor backward flow through Tesla-type bend. Global liquid supply in the whole channel length is significantly enhanced.</td></tr>
<tr><td>Copper Tesla channel configuration
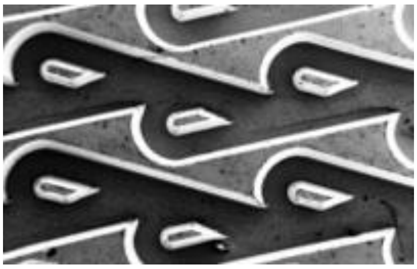</td><td>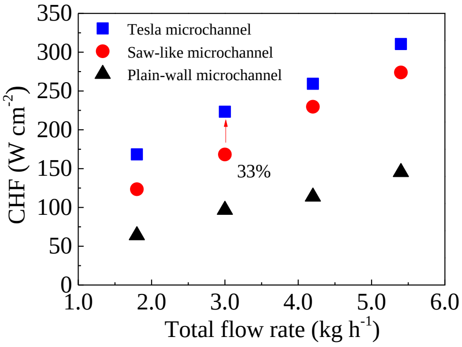

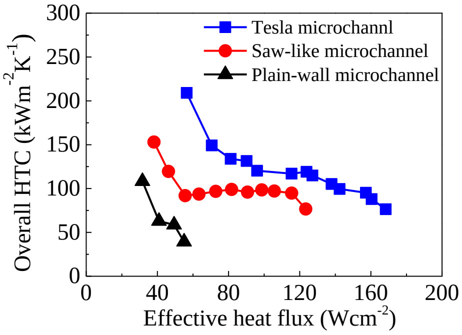
</td><td>CHF is further enhanced by ~33% with the integration of Tesla island. Once vapor backflow encounters Tesla island, one-third vapor backflow goes through the bend and then the backflow is rectified. The accumulated effect of Tesla valve leads to directional two-phase flow by rectifying vapor backflow.</td></tr>
</table>

3. Supplementary references:

1 Rui, Z., et al. Experimental research on flow boiling thermal-hydraulic characteristics in novel

microchannels. *Exper. Therm. Fluid Sci.* **140**, 110755 (2023).

2 Boreyko, J. B., et al. Planar jumping-drop thermal diodes. *Appl. Phys. Lett.* **99**, 234105 (2011).

3 Miljkovic, N., et al. Electric-field-enhanced condensation on superhydrophobic nanostructured surfaces. *ACS Nano* **7**, 11043-11054 (2013).

4 Cha, G., et al. Thermal conductance switching based on the actuation of liquid droplets through the electrowetting on dielectric (EWOD) phenomenon. *Appl. Therm. Eng.* **98**, 189-195 (2016).

5 McLanahan, A. R., Richards, C. D. & Richards, R. F. A dielectric liquid contact thermal switch with electrowetting actuation. *J. Micromechan. Microengin.* **21**, 104009 (2011).

6 Seshadri, I. *et al.* Gating heat transport by manipulating convection in a magnetic nanofluid. *Appl. Phys. Lett.* **102**, 203111 (2013).

7 Boreyko, J. B. & Chen, C.-H. Vapor chambers with jumping-drop liquid return from superhydrophobic condensers. *Int. J. Heat Mass Transfer* **61**, 409-418 (2013).

8 Tsukamoto, T., Hirayanagi, T. & Tanaka, S. Micro thermal diode with glass thermal insulation structure embedded in a vapor chamber. *J. Micromechan. Microengin.* **27**, 045001 (2017).

9 Ozkan, O., Shahriari, A. & Bahadur, V. Electrostatic suppression of the Leidenfrost state using AC electric fields. *Appl. Phys. Lett.* **111**, 141608 (2017).

10 Shahriari, A., Ozkan, O. & Bahadur, V. Electrostatic Suppression of the Leidenfrost State on Liquid Substrates. *Langmuir* **33 46**, 13207-13213 (2017).

11 Cho, H. J., Mizerak, J. P. & Wang, E. N. Turning bubbles on and off during boiling using charged surfactants. *Nat. commun.* **6**, 8599 (2015).

12 Li, W. *et al.* Supercapillary architecture-activated two-phase boundary layer structures for highly stable and efficient flow boiling heat transfer. *Adv. mater.* **32**, e1905117 (2020).

13 Zhu, Y. *et al.* Surface structure enhanced microchannel flow boiling. *J. Heat Transfer* **138**, 091501 (2016).

14 Krishnamurthy, S. & Peles, Y. Flow boiling of water in a circular staggered micro-pin fin heat sink. *Int. J. Heat Mass Transfer* **51** (2008).

15 Kuo, C. J. & Peles, Y. Local measurement of flow boiling in structured surface microchannels. *Int. J. Heat Mass Transfer* **50**, 4513-4526 (2007).

16 Palko, J. W. *et al.* Extreme two-phase cooling from laser-etched diamond and conformal, template-fabricated microporous copper. *Adv. Funct. Mater.* **27**, 1703265 (2017).

17 Ahmadi, M. & Bigham, S. Gradient wick channels for enhanced flow boiling HTC and delayed CHF. *Int. J. Heat Mass Transfer* **167**, 120764 (2021).

18 Li, W. *et al.* Enhanced flow boiling in microchannels through integrating multiple micro-nozzles and reentry microcavities. *Appl. Phys. Lett.* **110**, 014104 (2017).

19 Qu, W. & Mudawar, I. Flow boiling heat transfer in two-phase micro-channel heat sinks-I. Experimental investigation and assessment of correlation methods. *Int. J. Heat Mass Transfer* **46**, 2755-2771 (2003).

20 Jones, B. J. & Garimella, S. V. Surface roughness effects on flow boiling in microchannels. *J. Therm. Sci. Engin. Applicat.* **1** (2010).

21 Kalani, A. & Kandlikar, S. G. Combining liquid inertia with pressure recovery from bubble expansion for enhanced flow boiling. *Appl. Phys. Lett.* **107**, 181601 (2015).
