# Supplementary material for: Tesla valves and capillary structures-activated thermal regulator
Source: Nat Commun. 2023 Jul 6;14:3996. doi: 10.1038/s41467-023-39289-5 (PMC10325955; doi:10.1038/s41467-023-39289-5)
Supplement: Supplementary file 3 — Description of Additional Supplementary Files [file 41467_2023_39289_MOESM3_ESM.pdf]

Description of Additional Supplementary Files

File Name: Supplementary Movie 1
Description: Demonstration of the successful rectification of severe vapor backward in Tesla channels. $\dot{m}$ = 0.18 kg h $^{-1}$ and heat flux is 354 W cm^{-2}. In striking contrast, a persistent vapor backflow is observed in the control device without the presence of typical Tesla valves. This video is recorded by a high-speed camera of Phantom V7.3 at approximate 4,000 fps and replayed at 3 fps. The resolution is 1400 × 352 pixels.

File Name: Supplementary Movie 2
Description: Achieving a quick liquid refilling into Tesla channels by the successful rectification of severe vapor backward flow. $\dot{m}$ = 0.18 kg h^{-1} and heat flux is 354 W cm^{-2}. In the control device, liquid is incapable of entering the channels as imposed by a persistent vapor backflow. This video is recorded by a high-speed camera of Phantom V7.3 at approximate 4,000 fps and replayed at 3 fps. The resolution is 640 × 352 pixels.

File Name: Supplementary Movie 3
Description: Conformal capillary structures enable quick liquid rewetting to maintain a sustainable thin liquid film in the thermal regulator. $\dot{m}$ = 0.18 kg h^{-1} and heat flux is 500 W cm^{-2}. The rewetting velocity is ~0.5 m s $^{-1}$. Directional liquid flow is observed along sidewalls. This video is recorded with a high-speed camera of Phantom V7.3 at approximate 4000 fps and replayed at 3 fps. The resolution is 640 × 352 pixels.

File Name: Supplementary Movie 4
Description: Dynamic two-phase flow behaviors are controlled by the working state of the thermal regulator. When χ < 0.25, vapor backflow near the inlet plenum is observed at $\dot{m}$ = 0.23 kg h^{-1}. When χ > 0.25, directional two-phase flow is achieved the corresponding switched-on state. The duration of vapor backflow significantly decreases from 6 ms to 0.75 ms. This video is recorded with a high-speed camera of Phantom V7.3 at approximate 4000 fps and replayed at 3 fps. The resolution is 640 × 352
